# Public attitudes to, and behaviours taken during, hot weather by vulnerable groups: results from a national survey in England

**DOI:** 10.1186/s12889-021-11668-x

**Published:** 2021-09-06

**Authors:** Bob Erens, Lorraine Williams, Josephine Exley, Stefanie Ettelt, Tommaso Manacorda, Shakoor Hajat, Nicholas Mays

**Affiliations:** 1 Policy Innovation & Evaluation Research Unit, Department of Health Services Research & Policy, London School of Hygiene & Tropical Medicine, London, WC1H 9SH UK; 2 Department of Public Health, Environments & Society, London School of Hygiene & Tropical Medicine, London, WC1H 9SH UK

**Keywords:** Heat, Hot weather, Older people, Emergency planning

## Abstract

**Background:**

Hot weather leads to increased illness and deaths. The Heatwave Plan for England (HWP) aims to protect the population by raising awareness of the dangers of hot weather, especially for those most vulnerable. Individuals at increased risk to the effects of heat include older adults, particularly 75+, and those with specific chronic conditions, such as diabetes, respiratory and heart conditions. The HWP recommends specific protective actions which relate to five heat-health alert levels (levels 0–4). This study examines the attitudes to hot weather of adults in England, and the protective measures taken during a heatwave.

**Methods:**

As part of a wider evaluation of the implementation and effects of the HWP, a survey (*n* = 3153) and focus groups, a form of group interview facilitated by a researcher, were carried out after the June 2017 level 3 heat-health alert. Survey respondents were categorised into three groups based on their age and health status: ‘vulnerable’ (aged 75+), ‘potentially vulnerable’ (aged 18–74 in poor health) and ‘not vulnerable’ (rest of the adult population) to hot weather. Multivariable logistic regression models identified factors associated with these groups taking protective measures. In-person group discussion, focused on heat-health, were carried out with 25 people, mostly aged 75 + .

**Results:**

Most vulnerable and potentially vulnerable adults do not consider themselves at risk of hot weather and are unaware of the effectiveness of important protective behaviours. Only one-quarter of (potentially) vulnerable adults reported changing their behaviour as a result of hearing hot weather-related health advice during the level 3 alert period. Focus group findings showed many vulnerable adults were more concerned about the effects of the sun’s ultra-violet radiation on the skin than on the effects of hot temperatures on health.

**Conclusions:**

Current public health messages appear to be insufficient, given the low level of (potentially) vulnerable adults changing their behaviour during hot weather. In the context of increasingly warmer summers in England due to climate change, public health messaging needs to convince (potentially) vulnerable adults of all the risks of hot weather (not just effects of sunlight on the skin) and of the importance of heat protective measures.

**Supplementary Information:**

The online version contains supplementary material available at 10.1186/s12889-021-11668-x.

## Background

In England, periods of hot weather lead to increases in deaths and illness [1]. A severe heatwave in 2003 led to over 2000 excess deaths in England and Wales, especially in southern regions and among people aged 75+ [2]. With predictions of more frequent and increasingly hotter summers in England [3], accompanied by increasing numbers of people aged 75+, awareness of heat protection behaviours is of growing importance.

Since 2004, the government has implemented a Heatwave Plan for England (HWP) [4], which aims to protect the population by raising awareness of the dangers of severe heat, and recommends protective actions to be taken by National Health Service (NHS), local government, community groups and individuals [5]. The specific actions to be taken relate to five heat-health alert levels (levels 0–4). Levels 3 and 4 are triggered when specified temperature thresholds are reached. The temperature thresholds vary by region within England, but average to 30 C by day and 15 C overnight for at least two consecutive days. Additional file 1 shows the temperature thresholds for level 3 alerts for each region in the country. In 2017 a level 3 alert was issued for 3 days in June, and in 2018 there were three separate level 3 alerts issued, over July and August, lasting for seven, five and 1 day respectively.

Understanding risk, and how to adapt individual behaviours to prevent illness or death during hot weather, is especially important for those most at-risk to the effects of heat [6]. The HWP identifies factors that increase an individual’s risk during hot weather. These include age, particularly 75+, as ageing diminishes thermoregulatory function [7]; the very young; chronic or severe illness e.g. certain respiratory and heart conditions, diabetes; alcohol and/or drug dependency; homelessness; inability to adapt behaviour to keeping cool; and environmental factors (e.g. living in urban areas).

Results from surveys after level 3 heat-health alerts in England in 2013 showed that, despite widespread awareness of the effectiveness of most protective behaviours, many adults did not consider themselves to be at-risk and relatively few changed their behaviour during the hot weather [8, 9]. These findings may be partly explained by the positive feelings many UK residents hold towards warm summers, which tend to reduce perceptions of risk [10].

In 2016, the Department of Health and Social Care (DHSC) commissioned an independent evaluation of the implementation and effects of the HWP [11]. This paper presents findings from a survey and focus groups undertaken in England in 2017 and 2018 to examine the protective measures taken by the population in response to a specific heat-health alert in June 2017. It compares perceptions of the effectiveness of heat protection measures among three groups: older people aged 75+ (vulnerable group); adults aged 18–74 in poor health (potentially vulnerable group); and the rest of the adult population (not vulnerable). It also examines behaviour change during the level 3 heat-health alert between 16 and 23 June, which was associated with an estimated 666 excess deaths [12].

## Methods

### Survey

#### Survey development

The survey, modelled on two earlier surveys [8, 10], included questions on: whether respondents love hot weather and perceive it as a risk to their health (rated on a 5-point scale from strongly agree to strongly disagree); and perceived effectiveness of nine heat protection measures (rated on a 5-point scale from completely effective to not at all effective).

Individuals who were in England during the level 3 heat-health alert in June 2017 were asked about their experiences, including whether: they had heard heat-alerts and health advice; they had changed their behaviour as a result; and taken any heat protection measures (rated on a 5-point scale from never to always). Respondents were asked whether they had experienced any health effects as a result of hot weather. The survey questionnaire is shown in Additional File 2.

The questions were included in a larger survey conducted by the National Centre for Social Research (NatCen), which collected demographic data and questions on self-rated health (on a 5-point scale from very good to very bad) and whether respondents had any long-standing (i.e. anything that had lasted at least 12 months) physical or mental health condition that limited their ability to carry out normal activities.

#### Data collection

NatCen conducted the survey between 24 August - 24 September 2017 using its panel, which is representative of the population aged 18+ living at private residential addresses in England.

Participants were recruited using a sequential mixed mode design, with panel members first invited to complete the questionnaire online. Those who had not completed the survey online after 2 weeks, including those without internet access or those with language or literacy problems, were contacted by telephone to complete an interview. Respondents were sent a £5 gift card as a ‘thank you’, which is standard practice for surveys using the NatCen panel.

Of the 3153 panel members invited to participate, the achieved sample included 1633 online interviews and 245 telephone interviews, giving a response rate of 60%. Further details of the NatCen panel can be found in Jessop [13].

#### Analysis

Respondents’ vulnerability to hot weather was estimated based on their age, self-reported health status and whether they had a limiting long-standing illness (LLSI). Respondents were classified into three groups: (1) ‘not vulnerable’ (aged 18–74 with no underlying health condition); (2) ‘potentially vulnerable’ (aged 18–74 with an LLSI and/or in bad/very bad health); and (3) ‘vulnerable’ (aged 75+). Six respondents with insufficient information were excluded. Data were analysed in STATA (version 16.1).

Non-response weights were calculated to minimise any bias introduced by differential response among population sub-groups [13]. For each variable, percent frequencies and 95% confidence intervals (CIs) were calculated, weighted for non-response using the survey (svyset and svy) commands. Finally, multivariable logistic regression models were used to examine factors associated with vulnerable and potentially vulnerable individuals taking heat protection behaviours. Each heat protection behaviour was dichotomised as always/often versus occasionally/rarely/never. We present odds ratios (ORs) based on modelling each behaviour in a separate logistic regression model, which included: age; health (good/very good, fair or bad/very bad); LLSI (none, does not affect day-to-day life or affects day-to-day life); household type (living with someone or living alone); respondent’s perception of the effectiveness of the measure (completely/very effective or not/slightly/somewhat effective); perceived risk of hot weather to respondent’s health (strongly agree/agree, neutral or strongly disagree/disagree); whether respondent loved hot weather (strongly agree/agree, neutral or strongly disagree/disagree); whether they heard advice during the alert period (did not hear advice or heard advice); gender; education; household income; region; and location (urban or rural), weighted for non-response using the svy commands.

#### Focus groups

Four focus groups, discussing how older people cope in hot weather, were established in three areas in England, chosen to allow for variation in geography and exposure to heat. Participants were purposively selected to include those most at-risk during heatwaves: the majority were female (20 out of 25), all but one were aged 75+, and most lived alone and had health conditions vulnerable to heat. Participants were recruited through local luncheon clubs and voluntary organisations for older people. We used existing groups on the grounds that this would increase the likelihood of a richer discussion [14].

The topic guide covered attitudes towards hot weather, participants’ heat-health behaviours, and any identified risks and coping strategies. A selection of current health promotion leaflets/posters, such as the HWP’s ‘Beat the Heat’ campaign [15], were used as prompts. The topic guide is shown in Additional File 3.

Focus groups were audio-recorded and transcribed verbatim. A thematic analysis was undertaken using the Framework Method [16], whereby themes were identified, both deductively and inductively, from the research questions and participants’ narratives. A more detailed explanation of methods is available in [11]. Findings are interwoven with the survey results to provide contextual information for some of the responses in relation to vulnerable adults.

## Results

In total, 1872 respondents were included in the survey analysis, and 95% (*n* = 1787) reported being in England during the June 2017 level 3 heat-health alert period. Overall, 76% of the respondents were classified as ‘not vulnerable’ (group 1), 16% as ‘potentially vulnerable’ (group 2) and 8% as ‘vulnerable’ (group 3) to hot weather. Table 1 presents respondents’ characteristics. This section compares survey results between these three groups, supplemented by findings from the focus groups.

**Table 1:** Overview of survey respondents characteristics (weighted %)

	BSA England population estimate	All respondents (***n*** = 1872)	Not vulnerable (***n*** = 1422)	Potentially vulnerable (***n*** = 307)	Vulnerable (***n*** = 143)
**Age**	%	%	%	%	%
18–24	11	9	11	7	–
25–34	17	17	19	15	–
35–44	17	17	20	15	–
45–54	18	17	19	17	–
55–64	14	16	16	26	–
65+	22	28	15	20	100
**Gender**					
Male	48	48	47	47	56
Female	52	52	53	53	44
**Ethnic group**					
White	Not available	87	85	88	98
Other	Not available	13	15	12	2
**Highest educational attainment**					
Degree or equivalent	36	33	36	24	22
A level or equivalent	19	22	25	17	14
O level/CSE or equivalent	26	22	22	28	7
Foreign or other	3	8	7	9	18
No qualifications	17	15	10	23	39
**Monthly household income**					
Less than £1200	Not available	25	19	43	44
£1201 - £2200	Not available	25	24	25	33
£2201 - £3700	Not available	21	23	17	15
£3701 or more	Not available	29	34	15	8
**Region**					
North East	5	5	5	6	2
North West	13	13	14	11	11
Yorkshire & the Humber	10	10	9	10	15
East Midlands	9	8	8	10	10
West Midlands	10	10	9	10	14
East of England	11	11	11	13	12
London	16	15	16	15	5
South East	16	16	17	16	16
South West	10	11	10	9	16
**Location**					
Urban	Not available	82	81	86	75
Rural	Not available	18	19	14	25
**Household type**					
Single person household	17	17	12	26	37
Lone parent	4	4	4	3	0
2 adults (no children)	36	35	34	27	57
2 adults (with children)	21	23	26	20	0
3+ adults (no children)	15	16	17	19	5
3+ adults (with children)	7	6	7	5	0
**Social grade**					
Managerial/professional	38	41	43	30	37
Intermediate	12	14	15	12	11
Small employers/own account workers	9	8	7	10	8
Lower supervisory/technical	8	8	7	14	10
Semi-routine/routine	28	29	27	32	34
**Health**					
Good/very good	Not available	65	75	24	60
Fair	Not available	26	25	28	32
Bad/very bad	Not available	9	0	48	8
**Limiting longstanding illness (LLSI)**					
No	69	67	83	9	45
Does not affect day-to-day life	15	17	17	8	35
Affects day-to-day life	16	16	0	83	20

### Attitudes to hot weather

The majority of respondents reported they loved hot weather (57.7%), while less than a third identified hot weather as a risk to their health (31.1%) (Table 2). Even among the potentially vulnerable and vulnerable groups, fewer than half identified hot weather as a risk to their health, compared with half saying they loved hot weather.

**Table 2:** Attitudes, knowledge, and protective behaviours by type of vulnerable group

	All	Not vulnerable	Potentially vulnerable	Vulnerable
**Attitudes to hot weather [% of respondents that strongly agree or agree (95%CI)]**				
I love hot weather	57.7 (54.5–60.8)	60.4 (56.8–63.8)	49.9 (42.0–57.7)	48.7 (38.0–59.6)
Hot weather is a risk to my health	31.1 (28.3–34.1)	26.9 (23.9–30.0)	45.3 (37.6–53.4)	41.8 (31.0–53.5)
**Effectiveness of heat protection behaviours as [% of respondents rated behaviour very or completely effective (95%CI)]**				
Staying out of sun 11 am-3 pm	66.2 (63.0–69.1)	67.3 (63.7–70.6)	58.8 (50.7–66.5)	71.2 (60.3–80.1)
Drinking cool fluids	72.5 (69.6–75.3)	73.6 (70.3–76.8)	73.3 (65.6–79.8)	60.8 (49.0–71.5)
Covering skin with clothing	66.4 (63.2–69.4)	65.9 (62.2–69.4)	68.0 (59.8–75.2)	67.6 (55.7–77.6)
Limiting physical activity	64.2 (60.9–67.2)	64.5 (60.8–68.0)	62.3 (54.1–69.8)	64.8 (52.6–75.4)
Use electric fan	38.0 (34.9–41.2)	35.4 (31.9–39.0)	51.6 (43.8–59.4)	34.0 (24.1–45.6)
Close curtains on exposed windows	47.6 (44.5–50.8)	47.0 (43.4–50.6)	44.3 (36.7–52.2)	60.5 (49.2–70.7)
Close exposed windows during day	20.1 (17.6–22.8)	19.7 (16.9–22.8)	18.7 (12.7–26.6)	27.0 (19.0–36.9)
Open windows at night	63.1 (60.0–66.1)	63.9 (60.4–67.3)	58.7 (50.6–66.3)	65.1 (54.6–74.3)
Avoid alcohol	45.2 (42.0–48.4)	45.6 (42.0–49.3)	45.2 (37.5–53.2)	41.2 (31.1–52.2)
**Experienced adverse health effects as a result of hot weather or heat [% of respondents (95%CI)]**				
None	47.2 (44.1–50.3)	47.1 (43.5–50.7)	37.8 (30.7–45.4)	68.3 (56.7–78.0)
Dehydration/intense thirst	20.2 (17.6–23.0)	19.8 (17.0–23.0)	27.0 (19.6–35.8)	8.7 (4.8–15.1)
Sunburn	18.2 (15.6–21.0)	18.7 (16.0–21.7)	21.5 (14.4–30.8)	6.3 (1.3–24.8)
Heat rash/red and dry skin	14.0 (11.7–16.8)	12.5 (10.1–15.3)	22.3 (15.4–31.1)	11.1 (4.5–24.9)
Headaches	25.8 (23.0–28.8)	27.1 (23.8–30.7)	29.3 (22.8–36.8)	5.9 (3.0–11.4)
Dizziness	9.1 (7.3–11.2)	6.8 (5.1–9.0)	17.5 (12.5–23.9)	12.9 (5.6–26.7)
Nausea or vomiting	2.9 (1.8–4.5)	2.0 (1.2–3.3)	5.3 (2.2–11.9)	6.1 (1.2–25.5)
Muscle weakness or cramps	6.4 (4.9–8.4)	4.2 (3.0–5.8)	16.8 (10.9–25.1)	6.2 (2.9–12.6)
A high temperature	6.5 (4.8–8.6)	5.9 (4.1–8.4)	8.2 (5.2–12.8)	8.3 (2.5–24.3)
Irritability	21.3 (18.7–24.1)	19.2 (16.5–22.1)	33.5 (25.9–42.0)	15.2 (7.6–28.1)
A need to contact health services (e.g., a GP, an ambulance)	1.4 (0.6–2.9)	0.9 (0.3–2.3)	1.0 (0.3–4.0)	6.7 (1.6–24.4)
**Mean number of adverse health effects**	1.3 (0.1)	1.2 (0.1)	1.8 (1.5)	1.0 (0.3)
**Base (unweighted): All respondents**	**1872**	**1422**	**307**	**143**
**Heard and acted on health advice [% respondents (95%CI)]**				
Not heard advice	49.0 (45.8–52.2)	49.7 (46.0–53.5)	51.6 (43.6–59.5)	36.1 (26.4–47.1)
Heard advice but did not change behaviour	29.1 (26.3–32.0)	29.7 (26.5–33.1)	22.8 (17.4–29.4)	37.1 (26.3–49.4)
Heard advice and changed behaviour	21.9 (19.3–24.8)	20.6 (17.6–23.9)	25.6 (19.2–33.4)	26.8 (18.5–37.1)
**Protective behaviours taken [% respondents always or often took protective behaviours during 2017 heat alert period (95%CI)]**				
Staying out of sun 11 am-3 pm	46.6 (43.4–49.8)	44.8 (41.2–48.5)	48.6 (40.7–56.6)	59.2 (47.9–68.7)
Drinking cool fluids	87.3 (84.9–89.4)	88.6 (85.9–90.8)	84.8 (77.6–90.0)	80.6 (71.3–87.4)
Covering skin with clothing	50.9 (47.7–54.2)	49.3 (45.6–53.0)	51.7 (43.6–59.6)	65.0 (54.2–74.5)
Limiting physical activity	56.3 (53.1–59.6)	54.5 (50.7–58.2)	57.5 (49.2–65.4)	71.8 (60.4–80.9)
Use electric fan	39.3 (36.1–42.6)	38.6 (35.0–42.3)	46.3 (38.4–54.4)	31.0 (20.8–43.5)
Close curtains on exposed windows	47.7 (44.5–51.0)	46.5 (42.9–50.3)	49.6 (41.6–57.6)	55.3 (43.9–66.2)
Close exposed windows during day	34.9 (31.9–38.0)	33.2 (29.9–36.7)	34.3 (26.8–42.8)	52.4 (41.3–63.3)
Open windows at night	87.1 (84.5–89.3)	88.2 (85.1–90.8)	86.2 (80.3–90.5)	78.4 (69.1–85.5)
Avoid alcohol	51.2 (47.9–54.4)	47.7 (44.0–51.5)	61.1 (52.6–68.9)	62.8 (52.2–72.3)
**Mean number of protective behaviours always/often taken (sd)**	5.0 (2.1)	4.9 (2.1)	5.2 (2.0)	5.6 (2.2)
**Base (unweighted): In England during level 3 heat alert**	**1787**	**1356**	**295**	**136**

This attitude to risk was also found in the focus groups, where only a minority of participants said that they felt personally at-risk during heatwaves. In contrast with survey respondents, only a small number of focus group participants voiced positive attitudes towards hot weather, though any negativity expressed largely related to humidity rather than temperature. Participants were, on the whole, nonchalant about heatwaves, viewing them as a rare occurrence in England. *“We haven’t really had a summer”* was a common response.

### Views on the effectiveness of protective behaviours

The proportion of respondents who said the various heat protection measures were very/completely effective was relatively low, although this varied by group (Table 2). The not vulnerable and potentially vulnerable groups most frequently reported drinking cool fluids as very/completely effective (73.6 and 73.3%, respectively). Among the vulnerable group, staying out of the sun between 11 am and 3 pm was most frequently reported as effective (71.2%), and was also the behaviour most often mentioned by focus group participants. The behaviour least often considered very/completely effective, across all groups, was closing windows exposed to direct sunlight, ranging from 18.7% among potentially vulnerable to 27.0% among the vulnerable. Compared to other groups, the potentially vulnerable were the least likely to rate most behaviours as very/completely effective.

### Protective behaviours taken during the level 3 heat-health alert

While about half of all respondents said they were aware of hot weather-related health publicity or advice during the level 3 heat-health alert period (51.0%), this increased to about two-thirds in the vulnerable group (63.9%) (Table 2). However, even among the vulnerable, only 26.8% reported changing their behaviour as a result of hearing this advice.

Across all three groups, the heat protection measures respondents most frequently reported always/often undertaking were drinking cool fluids (87.3%) and opening windows at night (87.1%) (Table 2). The measures that the fewest respondents reported always/often taking were closing windows exposed to direct sunlight (34.9%) and using an electric fan (39.3%). Vulnerable respondents were the most likely to report taking six of the protective behaviours but were less likely to report using an electric fan, opening windows at night and drinking cool fluids. Although we cannot infer cause and effect in our survey, respondents in the vulnerable group were also the least likely to report any adverse health effects from hot weather in 2017: only 31.7% reported one or more health effects compared to 52.9% of the not vulnerable group and 62.2% of the potentially vulnerable group (Table 2).

Results of the multivariable logistic regression analysis focusing on potentially vulnerable and vulnerable groups combined are shown in Table 3. Although the small sample size (*n* = 431) limits the power to detect statistically significant results, there was strong evidence that respondents in these two groups were more likely to undertake a protective behaviour if they believed it to be completely/very effective. The odds ranged from more than twice as great for avoiding the sun (adjusted OR 2.6 [95%CI 1.5–4.5]) to more than nine times greater for closing exposed windows during the day (adjusted OR 9.6 [95%CI 4.1–22.5]). Those who reported being in bad/very bad health were more likely to avoid the sun (adjusted OR 2.1 [95%CI 1.0–4.3]), use an electric fan (adjusted OR 3.4 [95%CI 1.5–7.5]), close exposed windows (adjusted OR 2.2 [95%CI 1.0–4.8]) and avoid alcohol (adjusted OR 2.8 [95%CI 1.2–6.3]). Except for avoiding the sun, there was no difference between age groups. Having heard heat protection advice was only associated with closing exposed windows during the day (adjusted OR 1.7 [95%CI 1.0–3.0]) and opening windows at night (adjusted OR 2.2 [95%CI 1.1–4.5]), after controlling for other variables.

**Table 3:** Multivariable regression analysis examining heat protection behaviours always/often taken by potentially vulnerable and vulnerable groups (*n* = 431)

	Avoid sun OR (95%CI)	Drink cool fluids OR (95%CI)	Covers skin OR (95%CI)	Limits physical activity OR (95%CI)	Electric fan OR (95%CI)	Close curtain on exposed windows day OR (95%CI)	Close exposed windows day OR (95%CI)	Open windows night OR (95%CI)	Avoid alcohol OR (95%CI)
**Age**									
18–34	1	1	1	1	1	1	1	1	1
35–54	1.7 (0.7–4.2)	0.4 (0.0–5.5)	0.4 (0.1–1.1)	0.5 (0.2–1.6)	**0.2 (0.1–0.7)**	2.2 (0.8–6.2)	0.7 (0.3–1.9)	1.6 (0.4–6.2)	0.9 (0.3–2.8)
55–74	**2.5 (1.0–6.5)**	0.4 (0.0–5.8)	1.0 (0.3–2.7)	0.6 (0.2–1.9)	**0.3 (0.1–0.9)**	**3.2 (1.1–9.1)**	1.3 (0.5–3.6)	0.8 (0.2–2.9)	1.0 (0.3–3.0)
75 and over	**4.1 (1.2–14.7)**	0.3 (0.0–4.6)	0.8 (0.2–2.6)	2.2 (0.6–8.0)	0.4 (0.1–1.8)	1.7 (0.5–5.9)	1.4 (0.4–4.6)	0.3 (0.1–1.2)	2.0 (0.5–7.5)
**Health**									
Good/very good	1	1	1	1	1	1	1	1	1
Fair	1.8 (0.8–3.8)	0.8 (0.3–2.3)	1.2 (0.6–2.4)	1.3 (0.6–2.8)	**3.0 (1.3–6.8)**	1.4 (0.7–2.9)	**2.1 (1.0–4.5)**	1.3 (0.5–3.3)	**2.3 (1.1–5.2)**
Bad/very bad	**2.1 (1.0–4.3)**	0.6 (0.2–1.4)	0.8 (0.4–1.7)	1.4 (0.6–3.0)	**3.4 (1.5–7.5)**	1.5 (0.7–3.2)	**2.2 (1.0–4.8)**	0.8 (0.3–1.9)	**2.8 (1.2–6.3)**
**LLSI**									
No	1	1	1	1	1	1	1	1	1
Does not affect day-to-day life	**0.4 (0.1–1.0)**	1.2 (0.4–3.6)	0.7 (0.3–2.0)	0.7 (0.3–1.7)	0.5 (0.2–1.5)	0.8 (0.3–2.0)	0.8 (0.3–2.1)	0.8 (0.2–2.4)	1.2 (0.5–3.0)
Affects day-to-day life	0.8 (0.3–1.9)	0.9 (0.4–2.5)	0.4 (0.2–1.0)	0.9 (0.4–2.1)	0.6 (0.3–1.6)	0.7 (0.3–1.7)	) **0.3 (0.1–0.7)**	0.5 (0.2–1.4)	1.5 (0.6–3.7)
**Household type**									
Living with someone	1	1	1	1	1	1	1	1	1
Living alone	0.9 (0.5–1.7)	1.0 (0.5–2.3)	0.9 (0.5–1.7)	0.7 (0.4–1.4)	**0.4 (0.2–0.8)**	1.1 (0.6–2.2)	1.2 (0.7–2.4)	**0.5 (0.2–1.0)**	**0.4 (0.2–0.8)**
**Heard alert advice**									
Did not hear advice	1	1	1	1	1	1	1	1	1
Heard advice	1.4 (0.8–2.4)	1.9 (0.9–3.7)	1.1 (0.6–1.9)	1.0 (0.6–1.9)	1.3 (0.7–2.5)	1.0 (0.6–1.8)	**1.7 (1.0–3.0)**	**2.2 (1.1–4.5)**	1.4 (0.8–2.4)
**Effective**									
Not/slightly/somewhat	1	1	1	1	1	1	1	1	1
Completely/very	**2.6 (1.5–4.5)**	**6.2 (2.8–13.7)**	**2.8 (1.5–5.1)**	**3.7 (2.0–7.0)**	**4.9 (2.7–9.1)**	**6.5 (3.7–11.4)**	**9.6 (4.1–22.5)**	**3.7 (1.6–8.4)**	**4.3 (2.4–7.8)**
**Hot weather is a risk to health**									
Disagree/strongly disagree	1	1	1	1	1	1	1	1	1
Neutral	1.3 (0.6–2.6)	1.6 (0.6–4.7)	1.0 (0.5–1.9)	1.0 (0.4–2.2)	1.4 (0.6–3.2)	**2.2 (1.0–4.7)**	**2.5 (1.2–5.3)**	0.5 (0.2–1.2)	1.4 (0.7–2.8)
Agree/strongly agree	1.9 (1.0–3.7)	1.4 (0.5–3.9)	1.3 (0.6–2.7)	**2.1 (1.0–4.3)**	1.3 (0.6–2.7)	1.3 (0.6–2.7)	1.4 (0.6–3.0)	0.7 (0.3–1.6)	1.0 (0.5–2.0)
**Love hot weather**									
Disagree/strongly disagree	1	1	1	1	1	1	1	1	1
Neutral	0.8 (0.4–1.8)	1.3 (0.4–4.2)	1.0 (0.4–2.3)	0.5 (0.2–1.1)	**0.4 (0.2–1.0)**	**0.4 (0.1–0.9)**	1.2 (0.5–2.9)	**0.4 (0.1–1.0)**	0.9 (0.4–2.0)
Agree/strongly agree	0.8 (0.3–1.6)	1.2 (0.4–3.6)	**0.4 (0.2–0.9)**	0.6 (0.3–1.3)	0.9 (0.4–1.9)	**0.4 (0.2–0.8)**	1.1 (0.5–2.5)	0.6 (0.2–1.7)	0.5 (0.2–1.2)
**Sex**									
Male	1	1	1	1	1	1	1	1	1
Female	**1.9 (1.1–3.3)**	1.8 (0.9–3.8)	0.8 (0.5–1.5)	1.8 (0.9–3.4)	1.1 (0.6–2.0)	1.3 (0.7–2.3)	0.9 (0.5–1.6)	1.2 (0.5–2.6)	**1.9 (1.1–3.3)**
**Ethnicity**									
White	1	1	1	1	1	1	1	1	1
Other	2.1 (0.7–6.6)	0.4 (0.1–1.5)	0.3 (0.1–1.1)	1.7 (0.6–5.1)	1.1 (0.3–3.9)	1.3 (0.3–5.1)	1.2 (0.4–4.1)	0.7 (0.1–3.7)	4.4 (1.1–17.2)
**Education**									
Degree/higher	1	1	1	1	1	1	1	1	1
A-level/equivalent	0.4 (0.2–1.1)	1.6 (0.5–4.8)	0.6 (0.3–1.3)	0.3 (0.1–0.7)	1.9 (0.7–4.8)	0.7 (0.3–1.6)	0.7 (0.3–1.8)	1.7 (0.3–8.0)	0.5 (0.2–1.2)
Below A-level/none	0.5 (0.2–1.1)	1.4 (0.6–3.4)	0.7 (0.3–1.5)	0.6 (0.2–1.3)	1.9 (0.7–3.9)	0.5 (0.2–1.1)	1.0 (0.4–2.3)	0.5 (0.2–1.5)	1.1 (0.5–2.3)
Other	0.8 (0.3–2.3)	**4.9 (1.0–23.7)**	1.2 (0.4–3.9)	0.7 (0.2–2.3)	2.5 (1.0–6.5)	0.9 (0.4–2.4)	1.5 (0.4–4.9)	1.5 (0.3–7.5)	1.2 (0.4–3.6)
**Monthly household income**									
Less than £1200	1	1	1	1	1	1	1	1	1
£1201 - £2200	1.1 (0.6–2.3)	0.8 (0.3–2.0)	0.9 (0.5–1.8)	1.3 (0.6–2.9)	0.6 (0.3–1.3)	1.0 (0.5–2.1)	0.9 (0.5–1.9)	0.7 (0.3–1.7)	1.7 (0.8–3.7)
£2201 - £3700	1.5 (0.7–3.4)	1.8 (0.5–6.2)	1.7 (0.8–3.6)	1.9 (0.9–4.2)	1.2 (0.5–2.8)	0.8 (0.4–1.9)	0.6 (0.2–1.3)	1.3 (0.5–3.5)	0.7 (0.3–1.7)
£3701 or more	0.4 (0.1–1.1)	**5.0 (1.1–22.1)**	2.2 (0.8–6.0)	**3.0 (1.0–9.2)**	**2.8 (1.1–7.2)**	0.6 (0.2–1.6)	1.4 (0.5–3.9)	**6.8 (1.0–47.0)**	0.4 (0.2–1.2)
**Region**									
North	1	1	1	1	1	1	1	1	1
Midlands	1.3 (0.6–2.6)	1.9 (0.5–7.4)	0.8 (0.4–1.8)	1.1 (0.5–2.5)	**2.2 (1.0–4.9)**	1.5 (0.7–3.4)	0.6 (0.3–1.3)	0.8 (0.3–2.0)	1.1 (0.5–2.4)
East	0.8 (0.3–1.9)	0.6 (0.2–1.7)	0.6 (0.3–1.6)	1.5 (0.6–4.1)	1.9 (0.7–5.2)	1.4 (0.6–3.4)	0.6 (0.2–1.7)	0.7 (0.2–2.1)	1.3 (0.5–3.1)
London & South East	0.9 (0.4–2.0)	1.4 (0.5–3.8)	0.7 (0.3–1.4)	0.9 (0.4–2.1)	2.1 (1.0–4.4)	2.4 (1.1–5.3)	0.7 (0.3–1.5)	0.8 (0.3–2.2)	0.8 (0.4–1.6)
South West	1.8 (0.6–4.8)	1.0 (0.3–3.7)	0.7 (0.3–2.0)	1.0 (0.4–2.7)	1.0 (0.3–3.5)	1.2 (0.5–3.1)	0.4 (0.1–1.3)	**22.6 (2.1–248.1)**	2.5 (0.8–8.1)
**Location**									
Rural	1	1	1	1	1	1	1	1	1
Urban	0.9 (0.4–1.7)	1.5 (0.7–3.3)	0.9 (0.4–1.9)	1.0 (0.5–1.9)	1.4 (0.6–3.5)	0.8 (0.4–1.7)	1.9 (0.9–3.9)	1.3 (0.5–3.0)	1.5 (0.7–3.1)

*Note*: Each logistic regression model controls for all covariates shown in the table

Bold text *p* < 0.05

The focus group findings provide context to explaining behaviours taken, specifically those the survey identified as being *less likely* to be taken by vulnerable groups. Participants believed they had good knowledge of actions to take in a heatwave; it was, for them, *“common sense’*”, and few felt they belonged to any special risk category. Most said they stayed out of the sun and covered their skin. For many this was to avoid harmful effects of ultra-violet rays, as, according to some participants, ageing *“thinned the skin”*, thereby making it more susceptible to sun damage. Other measures, such as opening windows at night, drinking extra fluids, or using a fan, were inconsistently applied or absent. Risk awareness, security, and costs were identified as key reasons.

A strong theme was safety and security. Most participants kept their windows closed during hot nights for fear of intruders, opting to endure any short-lived discomfort and appearing unaware of any added risks to their health. Cost and noise were identified as reasons for not employing electric fans, particularly at night. Others spoke of their lack of knowledge of some protective measures, such as closing and shading sun-facing windows during the day, or not being aware of raised indoor temperatures, expressing surprise when visitors told them *“it’s really hot in here”*. Additionally, even though all agreed that maintaining hydration was important in hot weather, some admitted that they did not drink enough, yet were unconcerned about it as they *“did not often feel thirsty”*.

## Discussion

### Main findings

Heat protection messages often target older people and those in poor health because of their greater vulnerability to heat-related harm [17]. Our results show that many vulnerable and potentially vulnerable adults do not consider hot weather to be a risk to their health. They also illustrate that a person’s attitude to hot weather appears to shape their behaviour, including those in vulnerable and potentially vulnerable groups, which accords with results from previous studies among the general population [18–21]. Moreover, much current public health messaging does not appear to be having the desired effect. Only around a quarter of respondents in the potentially vulnerable and vulnerable groups reported changing their behaviour during the level 3 heat-health alert period in June 2017 as a result of having heard hot weather-related health advice.

The focus groups help interpret these survey findings. Individual narratives of personal health risks during heatwaves frequently referred to the effects of the sun’s ultra-violet radiation on the skin, rather than to the effects of hot temperatures on health. This has been noted in a previous study [18] and is reflected in current guidance provided by a national charity advising older people [22], which gives primacy to information about sun and skin health. As long as they stayed in the house, or in the shade, participants did not identify themselves as ‘at-risk’, and were less likely to take other actions to protect themselves from heat. This also explains why some felt it was inappropriate for them to be identified as ‘vulnerable’ by health and social care services, as in the HWP. These findings support those found in another recent study [23] showing older people being less likely than younger people to be aware of the risks of hot weather and taking appropriate actions.

For four of the nine heat protection behaviours examined, a majority of the potentially vulnerable and vulnerable groups were not aware of their effectiveness, including closing exposed windows during the day, closing curtains on exposed windows during the day (potentially vulnerable group only), using an electric fan and avoiding alcohol. Raising awareness is important as our results show that even people in the potentially vulnerable and vulnerable groups who recognised the risk of hot weather were much less likely to take protective measures if they did not consider the behaviours to be effective. This lack of awareness means many potentially vulnerable adults are not taking all the measures they should to protect themselves from the potentially harmful health effects of hot weather.

### Limitations of this study

For the survey analysis, apart from people aged 75+, we could only approximate which individuals may be vulnerable to adverse health impacts of hot weather. We used the term ‘potentially vulnerable’ to indicate that some members included in this group may not actually be at-risk since we do not know, for example, whether those reporting a LLSI had a chronic condition that increased their health risk during hot weather.

The study findings on behaviours taken and adverse health effects are based on self-reports and thus may be subject to mis-reports and recall bias, especially as the survey and focus groups took place several months after the June 2017 heat-health alert period. Another limitation is that the survey response rate was 60%, and it is possible that vulnerable adults in poorer health may have been less likely to complete the questionnaire, although corrective weighting was undertaken to limit the effects of non-response bias.

To limit sensitivity bias, focus group participants were instructed that they should only reference personal information that they felt happy to share within the group and were not prompted to do so. This may have led to some under-reporting of health or social conditions that might have influenced responses. However, as all groups were ‘natural’ groups, in that they met regularly through their luncheon or tea groups, participants appeared to speak freely about their health and social conditions during the interviews. As a result of the focus groups being ‘natural’ groups, it was not possible to balance the groups in terms of gender, and there were more female participants as a result.

## Conclusions

Previous research has shown that the majority of the population does not change behaviour as a result of receiving advice during heat-health alert periods. Those who have positive views about hot weather are less likely to perceive themselves to be at risk of hot weather and to take protective actions; and individuals are more likely to take actions they perceive to be effective. Our study demonstrates that these findings also apply to groups generally considered vulnerable or potentially vulnerable to the effects of hot weather, including people aged 75+. In particular, these individuals do not see themselves as at-risk in hot weather and are unaware of the effectiveness of a number of important heat protective behaviours. Even vulnerable individuals who are aware of the dangers of hot weather to their health are unlikely to take protective actions if they do not recognise their effectiveness. Moreover, public health messages should avoid labelling individuals as ‘vulnerable’ [20], when raising awareness of the risks of hot weather even among those who fit the definition. Also, since high proportions of potentially vulnerable members of the population are not aware of the effectiveness of a number of important heat protection measures, there remains considerable scope to increase the uptake of these measures.

## A. Additional files

**Additional file 1.** "Heat-health alert level 3 temperatures by region” provides a map showing the temperatures required for triggering a level 3 alert for each region in England.

**Additional file 2.** Survey Questionnaire “General population survey questions” shows the questions, response categories and skip instructions for the survey.

**Additional file 3.** Topic Guide “Focus Group Topic Guide” shows the topic guide used in the focus groups.

## Data Availability

The data used and/or analysed for this article are available from the corresponding author on reasonable request.

## Abbreviations

BSA: British Social Attitudes Survey
CI: Confidence interval
DHSC: Department of Health and Social Care
HWP: Heatwave Plan for England
LLSI: Limiting long-standing illness
NatCen: National Centre for Social Research
NHS: National Health Service
NIHR: National Institute for Health Research
OR: Odds ratio
UK: United Kingdom
