# Supplementary material for: Public attitudes to, and behaviours taken during, hot weather by vulnerable groups: results from a national survey in England
Source: BMC Public Health. 2021 Sep 6;21:1631. doi: 10.1186/s12889-021-11668-x (PMC8422617; doi:10.1186/s12889-021-11668-x)

**Heat-health alert level 3 temperatures by region**

Threshold maximum day and night temperatures for the Met Office National Severe Weather Warning Service (NSWWS) region.

**Maximum temperatures (°C)**

**NSWWS Region Day Night**

London 32 18

South East 31 16

South West 30 15

Eastern 30 15

West Midlands 30 15

East Midlands 30 15

North West 30 15

Yorkshire and Humber 29 15

North East 28 15


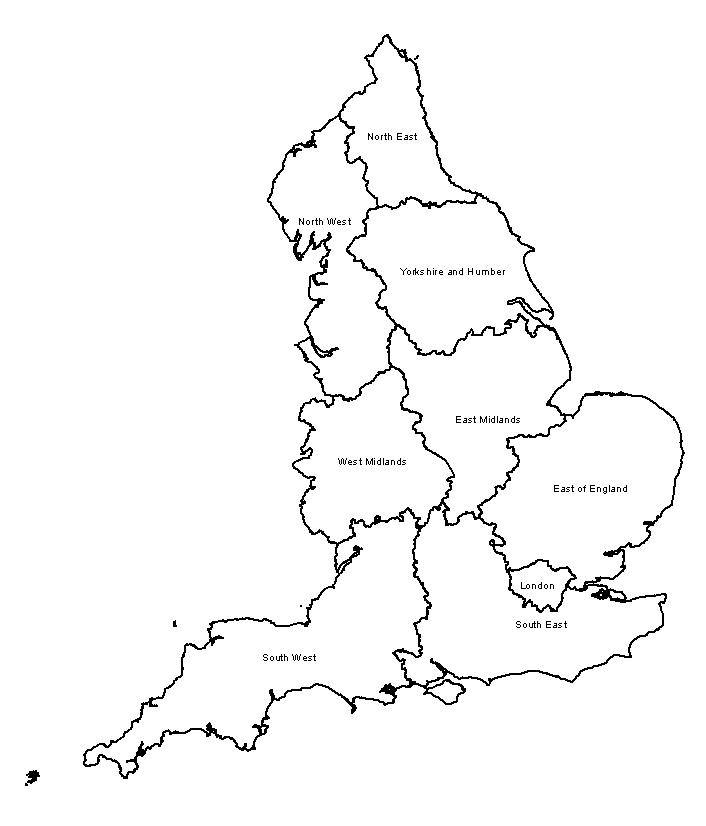

Supplement: Supplementary file 1 — Additional file 1. "Heat-health alert level 3 temperatures by region” provides a map showing the temperatures required for triggering a level 3 alert for each region in England. [file 12889_2021_11668_MOESM1_ESM.docx]
