# Supplementary material for: Public attitudes to, and behaviours taken during, hot weather by vulnerable groups: results from a national survey in England
Source: BMC Public Health. 2021 Sep 6;21:1631. doi: 10.1186/s12889-021-11668-x (PMC8422617; doi:10.1186/s12889-021-11668-x)
Supplement: Supplementary file 2 — Additional file 2. Survey Questionnaire “General population survey questions” shows the questions, response categories and skip instructions for the survey. [file 12889_2021_11668_MOESM2_ESM.docx]

## general population survey questions

{IF Cur_GOR <> 10...12} {IF living in England}

WeathAtt [GRID QUESTION: FLIP ANSWER SCALE; RANDOMISE ROWS; ONE PAGE]

The first set of questions is about hot weather.

To what extent do you agree or disagree with the following?

GRID ROWS:

1. I love hot weather
2. Spending time in the sun is good for me
3. Hot weather is a risk to my health
4. Hot weather is a risk to the health of someone I know
5. One good thing about climate change will be hotter summers in England

GRID COLS:

1. Strongly agree
2. Agree
3. Neither agree nor disagree
4. Disagree
5. Strongly Disagree

{IF Cur_GOR <> 10...12} {IF living in England}

EffecAct [GRID QUESTION: FLIP ANSWER SCALE; RANDOMISE ROWS; TWO PAGES]

How effective do you think the following actions are at protecting you from heat in the summer?

GRID ROWS:

1. Staying out of the sun between 11am and 3pm
2. Drinking cool fluids
3. Covering up your skin with clothing or using a hat
4. Limiting strenuous physical activity to the cooler parts of the day
5. Using an electric fan
6. Keeping curtains closed on windows exposed to direct sunlight during the day
7. Keeping windows closed that are exposed to direct sunlight during the day
8. Opening windows at night or in the cooler parts of the day
9. Avoiding alcohol

GRID COLS:

1. Completely effective
2. Very effective
3. Somewhat effective
4. Slightly effective
5. Not at all effective

{IF Cur_GOR <> 10...12} {IF living in England}

CoolLivSp

Are you able to keep your main living space at home cool during hot summer weather?

1. Yes
2. No

{IF Cur_GOR <> 10...12} {IF living in England}

CoolBedrm

And are you able to keep your bedroom at home cool during hot summer weather?

1. Yes
2. No

{IF Cur_GOR <> 10...12} {IF living in England}

Home [MULTICODE: RANDOMISE 1…6]

Does your home have any of the following?

MULTICODE

1. Roof or loft insulation
2. Cavity wall insulation
3. A ceiling fan in any room
4. Shaded areas outside your home (such as awnings or trees)
5. External shutters on any windows
6. White external walls
7. {#G_NoneAns_II1}: EXCLUSIVE

{IF Cur_GOR <> 10...12} {IF living in England}

InCountry

Most parts of England experienced hot weather in {June} this year between {17th and 21st June}, depending on the part of the country you live in.

Were you in England during that time?

1. Yes – in England on all or some of those days
2. No – out of England on all of those days

{IF InCountry = 1}

HeatYourHealth [GRID QUESTION: FLIP ANSWER SCALE; RANDOMISE ROWS; TWO PAGES]

During the heatwave/hot weather in {June}, how often, if at all, did you take the following actions to reduce any harm to <b>your health</b> from the heat?

GRID ROWS:

1. Stayed out of the sun between 11am and 3pm
2. Drank cool fluids
3. Stayed in the shade
4. Covered up your skin with clothing or wore a hat
5. Limited strenuous physical activity to the cooler parts of the day
6. Used an electric fan
7. Kept curtains closed on windows exposed to direct sunlight during the day
8. Kept windows closed that are exposed to direct sunlight during the day
9. Opened windows at night or in the cooler parts of the day
10. Avoided alcohol
11. Sought professional health advice

GRID COLS:

1. Never
2. Rarely
3. Occasionally
4. Often
5. Always

{IF InCountry = 1}

HeatOthersHealth [GRID QUESTION: FLIP ANSWER SCALE; RANDOMISE ROWS; TWO PAGES]

And how often, if at all, did you take the following actions to reduce any harm to the health <b>of others you know who are very young, or who are older, or who have a chronic/longstanding illness</b>?

GRID ROWS:

1. Ensured they stayed out of the sun between 11am and 3pm
2. Ensured they had cool fluids to drink
3. Ensured they stayed in the shade
4. Covered up their skin with clothing or a hat
5. Limited strenuous physical activity to the cooler parts of the day
6. Used an electric fan
7. Kept curtains closed on windows exposed to direct sunlight during the day
8. Kept windows closed that are exposed to direct sunlight during the day
9. Opened windows at night or in the cooler parts of the day
10. Avoided providing alcohol
11. Sought professional health advice

GRID COLS:

1. Never
2. Rarely
3. Occasionally
4. Often
5. Always

{IF InCountry = 1}

HeatPub

Were you aware of any hot weather-related health advice and publicity during this period of hot weather?

1. Yes
2. No

{IF HeatPub = 1}

HeatBehavCh

Did you change your behaviour in the hot weather as a result of this health advice and publicity?

1. Yes
2. No

{IF HeatPub = 1}

HeatAdvUse [FLIP ASNWER SCALE]

Would you say the health advice and publicity you heard was…?

1. Very useful
2. Fairly useful
3. Not very useful
4. Not at all useful

{IF InCountry = 1 AND (Cur_Age >= 65 OR FF_Age >=63 OR FF_Disability = 1)}

SpptVul [MULTICODE: RANDOMISE 1…5]

Which, if any, of the following people contacted you during the hot weather in {June} to check how you were?

MULTICODE

1. A GP or other doctor
2. A nurse or other NHS staff
3. Local authority/social services/social worker
4. Neighbour/friend/family member
5. Member of a voluntary organisation
6. Other (please describe)
7. INTERVIEWER: DO NOT READ OUT No-one contacted me: EXCLUSIVE

{IF Cur_GOR <> 10...12}

HeatIll [MULTICODE: RANDOMISE 1…10]

At any time during this summer, did you experience any of the following <b>as a result of hot weather or heat</b>?

MULTICODE

1. Dehydration/intense thirst
2. Sunburn
3. Heat rash/red and dry skin
4. Headaches
5. Dizziness
6. Nausea or vomiting
7. Muscle weakness or cramps
8. A high temperature
9. Irritability
10. A need to contact a GP or NHS 111, call an ambulance, or go to hospital or Accident & Emergency (A&E)
11. Other (please describe)
12. {#G_NoneAns_II1}: EXCLUSIVE
