# Supplementary material for: Public attitudes to, and behaviours taken during, hot weather by vulnerable groups: results from a national survey in England
Source: BMC Public Health. 2021 Sep 6;21:1631. doi: 10.1186/s12889-021-11668-x (PMC8422617; doi:10.1186/s12889-021-11668-x)
Supplement: Supplementary file 3 — Additional file 3. Topic Guide “Focus Group Topic Guide” shows the topic guide used in the focus groups. [file 12889_2021_11668_MOESM3_ESM.docx]

**Focus group topic guide**

General public (and carers of those) identified as vulnerable to heat

- Statement on purpose of study, anonymity and confidentiality.
- Statement on disclosure:

*Everything you tell me will be treated as confidential. However, should you mention something that leads me to believe that you and/or someone else is at risk of serious physical and/or emotional harm, I will have to pass this information on to* *the appropriate person*

- Answer any questions and check informed consents signed.
- Check consent for recording interview (will need to be consented by all – as in PIS)
- Agree aims and conduct of focus group
- Introductions (each to introduce themselves through facilitator)

**Questions:**

1. When we think about the weather, what would you say was ‘hot’?
2. What then is a heatwave?
3. Would you say that this is a problem in England? How frequent do they happen?
4. How do you know when we are expecting a heatwave [prompt – TV, radio, people, other?]
5. Can you name any health problems that are due to hot weather? [prompt if needed]
6. Who do you think is most at risk during a heatwave? Would you say you were at risk yourself? [prompt on heat health behaviour on holidays/travel abroad and whether any difference]
7. Have you ever received any advice/guidance about what to do in a heatwave? Tell us what you remember [prompt if needed – sunscreen, appropriate clothing, avoid going out midday, fluid intake, no strenuous exercise, close certain windows indoors, shade windows etc.]

- How was this advice provided (leaflets, coms from health/social care professionals/coms from family, friends/other?)
- Did you heed any of this advice? If not why? [prompt about any advice on heat abroad] Did you find any of it useful?

1. Do you think that your home gets too hot in the summer months?
   - If yes, have you tried to do anything to change this? What did you do? When was this done?
2. Is there anything you would like to do/change to make your home less hot but can’t do for some reason [e.g. cost/access/other]
3. Thinking back to the last time there was a long period of hot weather in England:

- Did you take any particular action? What did you do?
- Did you get any calls/visits from any health or social care practitioners during this time – if yes do you remember who called - what was said/done?
- Apart from close family/friends, did anyone else call/visit during this time (prompt local community group/neighbours/church reps) – if yes what was said/done?
- Do you think anyone should be doing more at this time [e.g. social workers, GPs, voluntary organisations] - if yes what would help?

Ask the group to indicate who has travelled to hot countries recently:

- [for those who have travelled to hot countries recently] How do you act here during hot weather compared to how you act abroad? (if differently) why do you think this is?

1. In this last ‘heatwave’ did you do anything differently to how you normally act/behave prior to and during a heatwave? – if yes – why was this?
2. Are you aware of [show beat the heat leaflets aimed at cohort] these leaflets?

If yes – where did you come across these? Do you heed their advice? If no – why not? Do you think they are helpful?

1. Finally what general advice would you give to protect yourself/others from severe hot weather?
